# The Association of Lactose Intolerance With Colon and Gastric Cancers: Friend or Foe?

**DOI:** 10.7759/cureus.24713

**Published:** 2022-05-03

**Authors:** Mohammad Maysara Asfari, Osama Hamid, Muhammad Talal Sarmini, Katherine Kendrick, Lakshmi Priyanka Pappoppula, Humberto Sifuentes, Subbaramiah Sridhar

**Affiliations:** 1 Department of Gastroenterology and Hepatology, Cleveland Clinic, Cleveland, USA; 2 Department of Hospital Medicine, Cleveland Clinic, Cleveland, USA; 3 Department of Gastroenterology and Hepatology, University of South Carolina, Greenville, USA; 4 Department of Gastroenterology and Hepatology, Augusta University Medical College of Georgia, Augusta, USA

**Keywords:** lactase, gastric cancer, colon cancer, national inpatient sample, lactose intolerance

## Abstract

Background: Lactose intolerance (LI) appears usually in later ages when the lactase enzyme becomes deficient or absent in the small intestine. Conflicting results have been reported in the literature about the association of lactose intolerance with various gastrointestinal malignancies. Hence, our aim was to study the association between LI, colon cancer (CCa), and gastric cancer (GC) using a large database. Methods: A cross-sectional study was performed using the National Inpatient Sample (NIS) database between 2004 and 2014. We identified adult patients (18-90 years) who were diagnosed with LI (study group) using appropriate International Classification of Diseases, Ninth Revision (ICD-9) codes. The control group comprised patients who did not have a diagnosis of LI. We identified the diagnosis of CCa and GC in both study and control groups using the ICD-9 codes. Univariable and multivariable logistic regression analyses were performed to assess the association between LI, CCa, and GC. Results: The total population comprised 71,360,501 patients, of which 57,909 (0.08%) were diagnosed with LI. LI patients were older (62 vs 51 years) with more females (61.5% vs 60.1%) and less African American patients (11.8% vs 14.3%) (p <0.0001 for all). In addition, LI patients had more smoking (12.4% vs 12%) and obesity (15% vs 8.9%). On the other hand, patients in the LI group had less alcohol use (3.8% vs 4.2%) (p <0.0001). After adjusting for the age, gender, race, smoking, alcohol, obesity, and inflammatory bowel disease, the LI group had a slightly lower rate of CCa (OR 0 .974, 95%CI 0.906-1.048, p = 0.486) and a lower rate of GC (OR: 0.993, 95%CI 0.924-1.068, p =0.853); however, the results were not statistically significant. Conclusion: Patients with lactose intolerance may have a lower risk of colon and gastric cancer. However, these findings were not statistically significant. Further studies are needed to understand this association.

## Introduction

Lactose is a disaccharide that is present in milk and dairy products. It is metabolized by an enzyme called lactase that is expressed from intestinal enterocytes, into glucose and galactose. Any decrease in the activity or deficiency of this enzyme results in a condition called lactose intolerance (LI). This can occur due to congenital absence of the enzyme, difference in the genetic polymorphisms, or secondary hypolactasia (as a result of damage to the mucosal brush border) [1]. The variations in the genetic polymorphisms of lactase enzyme results in two phenotypes - lactase persistence and lactase non-persistence [2]. Individuals who express lactase non-persistence phenotype are prone to developing LI. Studies have demonstrated that the expression of the lactase enzyme starts to decline after the weaning of breastfeeding. It has been hypothesized that with increasing age, there is down-regulation of the mRNA transcripts responsible for lactase enzyme expression [3]. As a result, LI is commonly encountered after younger years of life. Whenever individuals with LI consume milk and dairy products, the unabsorbed lactose is transmitted to the colon where it undergoes bacterial fermentation, resulting in the production of gases and short-chain fatty acids. These products not only cause colonic irritation but also alter the luminal osmolality in the bowel, thereby impairing water absorption in the colon. These factors contribute to the clinical features of LI, i.e. secretory watery diarrhea, abdominal pain, and bloating [2,4]. Colon cancer (CCa), prostate, and ovarian cancer are the areas where the protective and adverse effects of LI have been studied and investigated [5,6,7]. Several epidemiological studies on the association of dairy products and gastric cancer (GC) showed conflicting results on this relationship [8,9,10]. The protective effects of milk and dairy products are believed to be due to the components like calcium, vitamin D, conjugated linoleic acid, and sphingolipids. Calcium and vitamin D play a role in cell growth and differentiation by interacting with the calcium-sensing receptors, and calcium also helps in neutralizing the toxic effects of free fatty acids and bile acids by forming insoluble non-toxic calcium salts [11,12]. One of the blood group antigens named Thomson-Friedenreich (TF) antigen (galactose-β 1,3-N-acetylgalactosamine), is being investigated and studied to identify its role in the pathogenesis of CCa. TF antigen is normally concealed in the colonic mucosa, but its expression can be increased as a result of glycosylation abnormalities. Galactose produced as a result of lactose metabolism in the intestine, normally binds to lectin and inhibits the mucosal proliferation. However, the increased expression of the TF antigen affects the ability of galactose to bind lectins, thereby stimulating the proliferation of the colonic mucosa [13]. Although several studies were conducted in populations from different ethnic backgrounds, the correlation between LI and CCa and GC remains controversial. Studies in Hungarian and Finnish populations have shown an increased risk of CCa. However, the studies in Italian, British, and Spanish populations do not support such an association [3]. Due to these uncertain results, we aimed to study the relationship of LI with CCa and GC using a large national database.

This article was presented as an abstract poster at the Digestive Disease Week (DDW) May 18-21, 2019, in San Diego, California, and is available in the American Gastroenterological Association (AGA) official journal (abstract Su1970).

## Materials and methods

Data source

We conducted a cross-sectional study using National Inpatient Sample (NIS) data from 2004 to 2014. The NIS is the largest all-payer inpatient database in the United States and contains a database of over eight million inpatient stays each year, which represents approximately 20% of discharges from all community hospitals participating in the Healthcare Cost and Utilization Project (HCUP). It excludes rehabilitation and long-term acute care hospitals. Each record of the NIS database includes primary and secondary diagnoses up to 25, as well as primary and secondary procedures up to 15. It also contains patients' demographics, discharge status, length of stay, disease severity, and comorbidity measures.

Study population, inclusion, and exclusion criteria

We included all adult patients (≥18 years old) from the NIS database between the years 2004 and 2014. Using the International Classification of Diseases, Ninth Revision (ICD-9) code, we identified all patients with LI, CCa, and GC using the codes identified in Table 1. The HCUP comorbidity software was used to generate Elixhauser comorbidities from ICD-9 diagnosis codes.

**Table 1:** ICD-9-CM and CCS codes used to identify co-morbidities CCS: Clinical Classification Software; ICD-9-CM: International Classification of Diseases, Ninth Edition, Clinical Modification

Variable	Source	Code(s)
Lactose intolerance	ICD-9-CM	271.3
Colon Cancer	ICD-9-CM	153.0, 153.1, 153.2, 153.3, 153.4, 153.5, 153.6, 153.7, 153.7,153.8, 153.9
Gastric Cancer	ICD-9-CM	151.0, 151.1, 151.2, 151.2, 151.3, 151.4, 151.5, 151.6, 151.7, 151.8, 151.9
Inflammatory bowel disease	CCS	144
Smoking	ICD-9-CM	305.1

Study variables

Patients’ demographics and comorbidities were identified using the Clinical Classification Software (CCS) codes provided by the HCUP. Comorbidities of interest were defined by querying all diagnostic and procedural fields for the corresponding ICD-9 codes (Table 1 and Table 2). Patients were divided into two groups: patients with LI (study group) and patients without LI (control group). We then assessed the association of LI with CCa as well as GC and compared this to patients without LI. Because NIS is a publicly available database that includes de-identified patient data, Institutional Review Board (IRB) approval was not required.

**Table 2:** Elixhauser comorbidities included in the study

Elixhauser comorbidities
Alcohol abuse
Obesity

Statistical analysis

The data was expressed as mean values ± standard deviation, and frequencies were reported in percentages. Independent t-tests were used for the comparison of continuous variables measurements, while chi-square test was used for categorical variables. Multivariate logistic regression analysis was used to assess the association between LI and both CCa and GC. The regression model was adjusted for: patients' demographics and other relevant comorbidities including obesity, smoking, alcohol abuse and inflammatory bowel disease (IBD). P-value < 0.05 was considered statistically significant. IBM SPSS Statistics for Windows, Version 25.0 (Released 2017; IBM Corp, Armonk, New York, United States) was used for all statistical analyses.

## Results

The total population comprised 71,360,501 patients, of which 57,909 patients (0.08%) were diagnosed with LI. LI patients were older (62 vs 57 years) with more females (61.5% vs 60.1%) and fewer African American patients (11.8% vs 14.2%) (P<0.001 for all). In addition, LI patients had more smoking (12.4% vs 12%), obesity (15% vs 8.9%) and IBD (2.2% vs 0.8%) (p <0.001 for all) compared to the control group. On the other hand, patients in the LI group had less alcohol use compared to patients without LI (3.8% vs 4.2%) (p <0.001) (Table 3).

**Table 3:** Baseline characteristics comparison of lactose Intolerance and non-lactose intolerance patients LI: lactose intolerance; SD: standard deviation; IBD: inflammatory bowel disease

Variable	LI	Non-LI	P-Value
Age (mean±SD)	61.51 ± 19.95	57.05 ± 20.85	<0.001
Female (%)	61.5	60.1	
Race (%)			<0.001
White	75.9	69	
Black	11.8	14.2	
Hispanic	7.1	10.7	
Asian or Pacific Islander	2.1	2.4	
Native American	0.6	0.6	
Other	2.4	3.0	
Primary expected payer (%)			<0.001
Medicare	51.9	45.2	
Medicaid	9.1	15.1	
Private Insurance	32.1	30.6	
Self-Pay	3.8	5.3	
No Charge	0.4	0.5	
Other	2.8	3.3	
Median Household Income (%)			<0.001
0 to 25 percentiles	24.1	29.2	
26 to 50 percentiles	26.7	26.1	
51 to 75 percentiles	25.5	23.6	
76 to 100 percentiles	23.6	21.1	
Bed Size (%)			<0.001
Small	12.3	13.7	
Medium	25.6	25.1	
Large	57.9	61.2	
Location/Teaching Status (%)			<0.001
Rural	14.5	12.3	
Urban Nonteaching	40.4	41.1	
Urban Teaching	45.1	46.6	
Hospital Region (%)			<0.001
Northeast	23.8	19.4	
Midwest	25.5	22.8	
South	32.1	39.0	
West	18.7	18.8	
Obesity (%)	15	8.9	<0.001
Smoking (%)	12.4	12.0	<0.001
Alcohol (%)	3.8	4.2	<0.001
IBD (%)	2.2	0.8	<0.001

**Figure 1:**
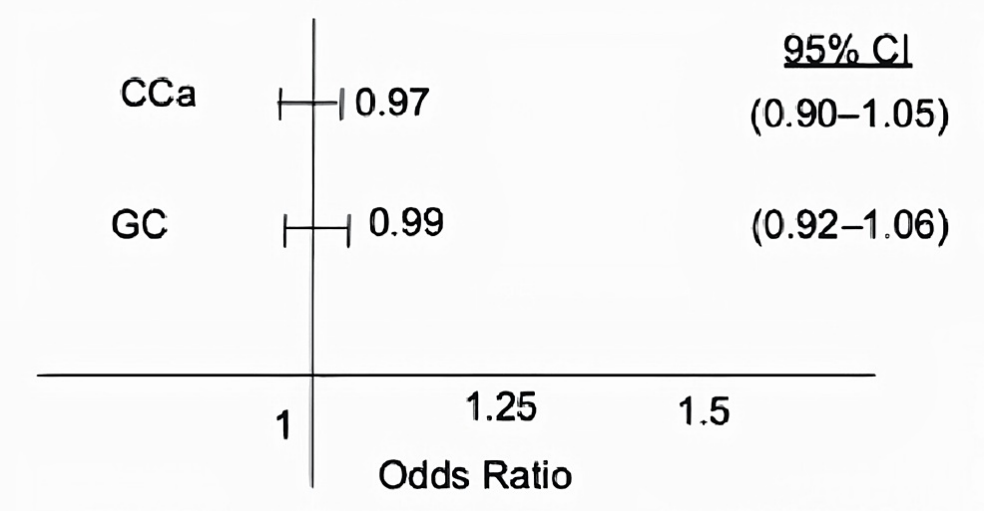
The odds ratio for the lactose intolerance association with colon cancer and gastric cancer CCa: colon cancer; GC: gastric cancer

Using multivariate logistic regression analysis and after adjusting for age, gender, race, smoking, alcohol, obesity and IBD, LI group did not have a statistically significant risk of CCa compared to patients without LI (OR: 0 .97, 95%CI 0.90-1.04, p = 0.48). Likewise, LI patients did not have a statistically significant risk for GC (OR: 0.99, 95% CI 0.92-1.06, p =0.85) (Figure 1).

## Discussion

Composed of a glucose and a galactose molecule, lactose is a carbohydrate in mammalian milk [14]. To allow for uptake by enterocytes in the intestinal wall, lactose is broken down via hydrolysis by the enzyme lactase-phlorizin hydrolase, which is found on the lactase gene (*LCT*) on chromosome 2q21 [15,16]. The hydrolyzed lactose forms glucose and galactose molecules, that can be oxidized to CO2 and H2O or, if under anaerobic conditions, undergo lactic or alcohol fermentation [14,17]. In the intestine, lactose becomes lactic acid, which lowers the pH of the gastric contents. The lowering of the pH of the gastric contents leads to alteration of the microflora composition, promotes beneficial bacteria, and increases the availability of absorbable calcium contents [14,18]. The bacteria promoted by alterations due to lactose ingestion cause regeneration of intestinal epithelial cells and promote the synthesis of short-chain fatty acids [14,19,20]. The digestion of lactose is essential for life. The expression of the *LCT* gene is genetically determined, although the activity of the enzyme is always highest in infancy [15]. In northern European populations, high levels of lactase enzyme can be maintained into adulthood, labeled lactase persistence [16]. Lactase-phlorizin hydrolase absence or underproduction leads to LI [14,21,22,23,24]. Four types of LI exist: congenital, developmental, secondary, and primary. Congenital is an autosomal recessive disease caused by a complete lack of lactase production [14,21]. The developmental type occurs when infants are unable to produce the enzyme at a sufficient level, due to premature birth [21]. Secondary intolerance is caused by intestinal villi injury, which can be secondary to viral or bacterial infections, parasites, enteropathy, or chemotherapy [14,21]. Lastly, the most common form of LI; primary lactase deficiency, occurs due to a genetically regulated decrease in lactase enzyme activity with age [14]. When lactose is not enzymatically broken down and absorbed by the enterocytes in the small intestine, negative effects on the intestine may result [25]. Undigested lactose is an osmotically active disaccharide and leads to the movement of water into the lumen of the gut, acidification of the lumen due to fermentation of the molecule by colonic bacteria, and alteration in the diversity of the intestinal bacteria [21,25]. This causes an increase in hydrogen (H2), carbon dioxide (CO2), and fatty acids, which potentiates an increase in stool volume, causing the symptoms of lactase deficiency [21]. The symptoms tend to occur approximately 30 minutes after ingestion of lactose and include, but are not limited to, the following: nausea, gas, diarrhea, abdominal pain, headaches, concentration disturbance, chronic rhinitis, sinusitis, and heart arrhythmias [14,22,23,26,27]. The lactose breath test is a non-invasive and inexpensive way to test for lactose intolerance. Other diagnostic methods include small intestine tissue biopsy and genetic testing of the C/T-13910 polymorphism [21]. Primary intolerance results from alterations via single nucleotide polymorphisms in the DNA sequence that codes the *LCT* gene at loci LCT-13910T>C [15,16,25]. The activity of LCT is broken down into three variants: the CC, CT, and TT genotypes. CC is for lactose malabsorbers; lactose absorbers are identified by CT and TT [15]. Ethnicity has an association with primary intolerance, with a high prevalence in American Indians, Asians, African Americans, Arabs, and Latin Americans [21]. The ability to identify and compare primary lactose-intolerant subjects and lactose-persistent subjects has provided a body of research for the establishment of diseases in those with and without lactase-phlorizin hydrolase. Numerous published studies have examined the protective or adverse effects of dairy on colon, ovarian, and prostate cancers. For some time, those studies have produced mixed results [5,6,7,21]. Our study also noted inconclusive results: patients with LI appeared to have a lower risk of CCa and GC; however, this finding lacked statistical significance. CCa is the second-most common cause of fatal cancer in the United States, which has led to extensive research on causation and prevention [21]. Research has found that diet has a direct association with CCa [12,21]. The World Cancer Research Fund/American Institute for Cancer Research and Aune et al. noted that milk, but not cheese (due to saturated fat), may have a protective effect on CCa [12,28,29]. That raises the question, does lactose intolerance contribute to the development of CCa? Studies among primary lactose-intolerant subjects with the CC genotype (low LCT activity) in Finnish populations have found an increase in CCa [15,30,31], while other studies examining Italian, British, and Spanish populations have not [15,21,30]. Szilagyi et al. performed a meta-analysis of various populations and determined that dairy products have a protective effect against CCa in populations with high or low lactose non-persistent subjects with high or low frequencies, but not in significant mixed lactose non-persistent/lactose persistent composition [32]. Although the incidence of GC has been declining worldwide, it remains an important cause of cancer-related mortality. Tian et al. in their meta-analysis examining the association of dairy products to GC found no clear association [8]. However, Sun et al.'s meta-analysis found a non-significantly increased risk of GC with the consumption of dairy products [9]. On the other hand, Guo et al.'s meta-analysis interestingly found a possible protective effect of dairy product consumption on GC on subgroup analysis of cohort studies; whereas case-control studies provided no association [10]. Research revealed that, as mentioned previously, milk may be protective, most likely related to the calcium and vitamin D in milk. Calcium and vitamin D have been shown to stimulate calcium receptors, causing cell growth and colonic epithelial differentiation in the intestine. Calcium, in the intestine, can also reduce the cytotoxicity of secondary bile acids and complex fatty acids [7,11,12,21,33], as well as protect against adenomatous polyps. It is important to note that studies that rely on reported dairy intake are difficult to interpret, because dairy products contain calcium as well as saturated fat, and the latter, theory suggests, is associated with an increased risk of CCa [12]. Milk products conjugated by colonic lactobacilli produce, in addition to calcium and vitamin D, conjugated linoleic acid, sphingolipids, and butyric acid, which are thought to have protective effects against CCa [7,16]. A study by Barton et al. found a decrease in colonic adenomas by 20% with a 1200 mg calcium supplement, with a 45% reduction in advanced adenomas. Sub-analysis of these findings revealed the highest benefit in patients with serum 25-hydroxy vitamin D levels of approximately 29 ng per mL [7,34,35]. Vitamin D intake has also been examined and revealed a 20% reduction in CCa [7,36]. The pathophysiology of the antitumor nature of vitamin D3 is poorly understood, but the anti-inflammatory effect of vitamin D may contribute [37]. Vitamin D increases calcium absorption in the intestine. Thus, when dairy food's effects on colon, ovarian, and prostate Cancers are under discussion, sun exposure should be under consideration [7,21]. Ovarian and prostate cancers, which were not examined in our study, are thought to be influenced by lactose because of its breakdown product, galactose. Excess galactose and estrogens in cow’s milk are believed to cause a toxic effect on germ cells in the ovaries. In one study, patients with high lactose consumption showed a two-fold elevated risk for serious subtypes of ovarian cancer; an 11g increase in lactose was associated with a 20% higher risk for ovarian cancer. The association of consumption of dairy products with prostate cancer, though controversial, may be influenced by the estrogens of cow’s milk in theory. The estrogens are believed to alter the growth of cells sensitive to estrogen, thereby possibly contributing to both prostate and ovarian cancers. However, studies have shown that the levels of estrogens in milk products are below the range determined to have an effect on consumers [21].

This study had some limitations. Due to ICD-9 limitations, NIS cannot confirm if the diagnosis of LI was proven by testing. Moreover, the NIS database depends on the precision of clinical data and the accuracy of medical diagnoses, which might differ among providers and facilities. The fact that NIS is based on inpatient data could have skewered the population towards a larger number of sick individuals in the data, which might have affected the generalizability of the results. Furthermore, the exclusion of academic hospitals from the NIS directory could potentially exclude patients with more complex diseases. However, the large nationwide database increases the power of the study, leading to potentially reliable conclusions.

## Conclusions

In conclusion, the findings from this large nationwide database study revealed that patients with LI may have a lower risk of CCa and GC. This inference is more suggestive rather than decisive given the insignificant statistical results. Although using LI as a surrogate to evaluate the milk effect on GI malignancies is potentially helpful, the overall picture is complicated and the pathophysiology behind the true effect remains to be fully understood. Further studies are needed to better understand the link between LI and CCa and GC.
